# Can Mixed Intercropping Protect Cereals from Aphid-Borne Viruses? An Experimental Approach

**DOI:** 10.3390/insects13060521

**Published:** 2022-06-04

**Authors:** Sarah Grauby, Aurélie Ferrer, Vincent Tolon, Anthony Roume, Alexander Wezel, Emmanuel Jacquot

**Affiliations:** 1 ISARA, Agroecology and Environment Research Unit, 23 Rue Jean Baldassini, 69364 Lyon, France; aferrer@isara.fr (A.F.); vtolon@isara.fr (V.T.); aroume@isara.fr (A.R.); awezel@isara.fr (A.W.); 2 PHIM Plant Health Institute Montpellier, University of Montpellier, INRAE, CIRAD, Institut Agro, IRD, CEDEX 5, 34398 Montpellier, France

**Keywords:** BYDV-PAV, *Rhopalosiphum padi*, morph, arena experiment, companion plant, clover, barley

## Abstract

**Simple Summary:**

Aphids are vectors of plant viruses and can cause important yield losses in numerous crops. *Rhopalosiphum padi* is the main vector of Barley yellow dwarf virus PAV (BYDV-PAV). The yellow dwarf disease (YDD), present in all cereal growing regions in the world, caused by BYDV-PAV, can induce up to 80% of yield losses in barley. In a context of global warming, reduction of pesticide uses, and development of organic farming, new control methods for YDD incidence are needed. In this study, the association of a winter barley with clover was tested for its impact(s) on the *R. padi*/BYDV-PAV pathosystem. The effect of clover was different according to the morph of the aphid introduced in a multi-plant (i.e., arena) experimental design in laboratory. A spatial effect on aphid distribution (observed for alate founder morph) and a reduction of the size of aphid populations (observed for wingless founder morph) are described. However, the presence of clover did not modify, under our experimental conditions, the efficiency of BYDV-PAV infections and within arena spread of YDD. Thus, clover used by cereal growers for other services (weeds reduction, nitrogen supply, and soil cover), would participate to lower the risks associated to the presence of BYDV vectors through a bottom-up regulation of aphid populations within the field. Barley/clover intercropping should be considered as a promising method for a future management strategy against vectors of yellow dwarf disease, especially in an insecticide-free agriculture.

**Abstract:**

Intercropping, i.e., association of two or more species, is promising to reduce insect populations in fields. The cereal aphid *Rhopalosiphum padi,* a vector of the Barley yellow dwarf virus PAV (BYDV-PAV), represents a major threat for cereal grain production. In this study, we tested the potential of winter barley intercropped with clover to reduce the size of *R. padi* populations and to lower the BYDV-PAV incidence in fields. We used arenas (i.e., sets of 36 barley plants) intercropped with or without clover plants (at different sown densities). In each arena, a single viruliferous founder, *R. padi*, (with an alate or a wingless morph) was deposited to introduce aphids and viruses in the experiment. Thirteen days later, the number of aphids in the arena, the percentage of plants hosting aphids and the infection rates were monitored. Data produced through this experimental design showed that clover alters the distribution of the aphid progeny (lower aphid spread) produced by an alate founder morph. Moreover, clover reduces the size of aphid populations produced by a wingless founder morph. However, despite the effects of clover on biological parameters of *R. padi*, the presence of clover in barley arena did not modify BYDV infections, suggesting complex mechanisms between partners of the BYDV pathosystem for plant-to-plant virus spread.

## 1. Introduction

Yellow dwarf disease (YDD) is one of the most important viral diseases in cereal crops worldwide, causing dwarfing, leaf discoloration, and yield losses up to 80% [1,2]. The disease is caused by a complex of ten virus species, transmitted in a persistent non-propagative manner by aphids [3]. At least twenty-five aphid species are involved in the epidemiology of YDD [4], and each of the ten virus species has its primary and secondary vectors species. In France, the bird cherry-oat aphid *Rhopalosiphum padi* (L.) (Hemiptera:Aphididae) is considered to be the main vector of YDD. Indeed, it is able to efficiently transmit BYDV-PAV, the most prevalent and damaging virus species reported in French cereal fields so far [5]. As many aphid species, *R. padi* exhibit wing dimorphism at different stages of its life cycle. In autumn, primary infections occur when viruliferous alate *R. padi*, whose flight threshold is 10 °C [6], introduces the virus into newly sown winter cereal fields. Then, when temperatures are above 5 °C, wingless individuals produced by parthenogenesis move from plant to plant and spread the virus within the field [7]. This step of the epidemical cycle corresponds to the secondary infections. When cereals are at an early development stage, they are highly susceptible to YDD. Thus, yield losses induced by YDD in winter cereals are mainly due to autumn infections [8,9]. As winter approaches, sexual alate individuals are produced and meet on the primary host of *R. padi*, i.e., *Prunus padus*, a small tree non-host for YDD. They form eggs which are more resistant to cold, and that will be the source of new spring populations [10]. However, in regions with mild winters, survival in egg form for aphids is less advantageous and reproduction in an asexual form (parthenogenesis; i.e., anholocyclic populations) continues throughout the cereal growing season. Global warming in temperate regions may impact the epidemiological cycle of YDD through the increase of anholocyclic aphid populations [11], the presence of aphids in cereal fields over a longer period, and the increase of virus spread. Thus, the damage related to YDD is expected to increase in the coming years.

Control methods used by farmers against YDD are limited. Cultural methods, such as late sowing and removal of volunteers, could constitute good options to limit the incidence of YDD on winter cereals [12]. However, mild autumns, which are increasingly frequent in a context of global warming, decrease the efficiency of late sowing. In conventional farming, insecticides were mainly used to control YDD. However, since the ban of neonicotinoids at the end of 2018, only pyrethroid-based foliage treatments remain with variable efficiencies. Thus, in the context of (i) the reduction of the use of plant protection products in cropping systems and (ii) the global warming, it is necessary to find new methods or practices to reduce aphid populations and YDD incidence.

The agroecological transition proposes new practices, relying more on natural processes and less on synthetic inputs. Agroecosystem diversification is one of the two pillars of this transition [13] and proposes to insert practices based on plant diversification at different scales. Among these practices, plant species association (i.e., intercropping) has been shown to bring many benefits for disease and pest managements. Concerning aphids, numerous studies show a decrease in their populations in intercropping systems compared to monocultures [14,15,16,17,18,19]. This biological control can be explained by top-down (indirect effect of the non-host plant on aphids via natural enemies) and bottom-up (direct effect of the non-host plant on aphids) mechanisms. In the bottom-up mechanisms, the non-host plant may interfere at different stages of the selection of host plants by aphid. Firstly, the non-host plant can interfere with different stimuli (olfactory [20,21], visual or physical [22]), that the aphid uses to detect its host plant. Then, if an aphid inadvertently arrives/lands on a non-host plant, it will start to make brief shallow test probes with its stylets [23] to evaluate the quality and the host/non-host status of the plant. The aphid can thus waste time testing a plant on which it will not feed and reproduce. Furthermore, once the host plant is found, the aphid can spend less time on it in an intercropping situation, as it has been shown in a recent study (up to 40% reduction of time spent) [24]. The main consequences of a shorter period spent on host plants by aphids are the production (i) of fewer offspring and (ii) milder direct (through sap removal) and/or indirect (through aphid-mediated transmission of viruses) damages. Thus, at field scale, one of the expected consequences of intercropping is a decrease of aphid populations and a lower aphid-mediated transmission of viruses. However, the impact of intercropping on the epidemiology of viral diseases has been poorly studied. Indeed, authors generally suggest that the decrease in aphid populations in an intercropping area probably leads to a decrease in the disease incidence. Hooks et al. [25] reviewed the effect of intercropping on viruses transmitted by aphids in a non-persistent manner and propose four potential mechanisms explaining the reduction of disease incidence. Concerning viruses transmitted in a persistent manner, which is the case for BYDV-PAV, studies on the impact of intercropping on viral epidemiology are lacking.

In this study, we chose clover as a non-host plant to disrupt *R. padi* and BYDV-PAV colonization of barley fields. This companion plant is already widely used in association with crops by organic farmers for many other services like nitrogen supply to the following crop, weeds control, and soil cover after the cereal harvest [26,27]. To accurately describe the impact of clover on epidemiological parameters involved in the introduction and spread of YDD in fields, lab experiments with pots containing barley plants, in the presence or absence of clover, were used and monitored over several weeks. Moreover, two clovers densities were tested. The following hypotheses were formulated:

1. The *Rhopalosiphum padi* total abundance in a multiple plants system is decreased in the presence of clover.
2. The BYDV-PAV incidence in barley is reduced in the presence of clover in arena.
3. The spatial distributions of aphids and virus in arena are modified in the presence of clover.
4. The effects of clover are more important for wingless founder aphids than for alate founder aphids.

## 2. Materials and Methods

### 2.1. Plants, Insects and Virus

**Figure 1:**
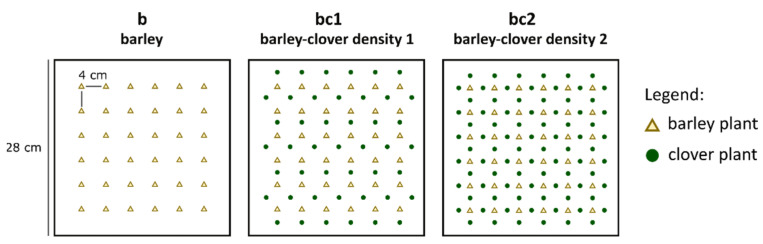
Experimental treatments sown for the arena experiment: barley (b) with 36 barley plants; barley-clover density 1 (bc1) with 36 barley plants + 45 clover plants; barley-clover density 2 (bc2) with 36 barley plants + 84 clover plants.

Barley seeds (cv. Etincel, [Sécobra France]) were sown in N2 soil (Neuhaus^®^ Huminsubstrat N2, klasmann Deilmann, Geeste, Germany) in square pots (L × W × H: 28 × 28 × 7 cm) in the presence or the absence of clover seeds, *Trifolium incarnatum* (cv. Viterbo). A perforated plexiglass seeding plate was designed to sow the different seeds (barley and clover) at specific locations in pots (Figure 1). Pots contain 36 barley seeds (6 rows × 6 lines) with a 4 cm distance between each seed. Two clovers densities were tested because the efficiency of the barley/clover association could be dependent on the density of clovers in the arena. Square pots containing only barley seeds are noted as b in the manuscript. The bc1 and bc2 square pots referred to pots containing barley plantlets associated to 45 and 84 clovers, respectively (Figure 1). Barley and clover seeds were sown to a depth of 2 cm and 1 cm, respectively. Six days after sowing, the germination of the barley seeds was checked, and missing plantlets were replaced by extra plantlets produced under the same conditions. A similar checking and replacement procedure was applied to clover plants but with an expected germination threshold of 80% per pot, leading to a minimum of 36 clovers in bc1 and 68 clovers in bc2. Pots were maintained in a growth chamber (day/night: 16 h/8 h; 24 °C) from sowing to the end of aphid exposure.

The *Rhopalosiphum padi* clone RpIA (collected in 2012 in the Yonne department, France) was used in the experiment. Viruliferous RpIA were reared on barley cv. Etincel plants infected by the isolate 4 of BYDV-PAV (BYDV-PAV4; [28]) in a small plexiglass cage maintained in a growth chamber (day/night: 16 h/8 h, 25 °C/20 °C; RH: 40%).

### 2.2. Arena Experiment

Square pots (b, bc1 and bc2, Figure 1) with clovers and/or barley plantlets were used to run arena experiments. Six days after sowing, a third instar nymph from a viruliferous RpIA population was deposited at the base of the plant located at the row 3 and line 3 of the arena using a fine brush (deposit tiller). The morph (wingless or alate) of the third instar nymph, called the founder aphid, was not known at the time of deposit. The arena was covered by an insect-proof net. This experimental set-up was maintained in a growth chamber for 13 days. At the end of this period, the number of aphids per barley plant was counted. Barley plants maintained in the square pots were sprayed with insecticide (Pirimor^®^ 0.1% *v*/*v*, Syngenta^®^, Basel, Switzerland). They were maintained in an insect-proof greenhouse for 3 weeks, the time at the end of which viral accumulation in the plant is maximal if the plant has been inoculated by aphids. At the end of this period, the sanitary status (presence of BYDV-PAV) of each barley plant was evaluated by an Enzyme-Linked Immuno-Sorbent Assay (ELISA). Then, 24 replicates per treatment over 4 sessions were planned (i.e., 6 replicates per treatment per session). Due to technical reasons (i.e., arenas lost because death of the founder aphid; very poor clover emergence), 65 arenas in total were finally run.

### 2.3. Serological Detection of BYDV-PAV in Plants

Each barley plant (all leaves) was individually sampled and grounded with a Pöllhane press (MEKU^®^, Wennigsen, Germany). The presence of BYDV-PAV in barley plants was tested using a double antibody sandwich enzyme linked immunosorbent assay (DAS-ELISA) (Clark and Adams, 1977). Wells of a microtiter plate (NUNC, Maxisorp, Thermo Fisher Scientific, Waltham, MA, USA) were incubated at 37 °C for 3 h with a polyclonal anti-BYDV-PAV antibody (PAV52, H. Lapierre, INRAE) previously diluted (1/1500 (*v*/*v*)) in a carbonate buffer (15 mM Na_2_CO_3_, 35 mM NaHCO_3_, pH = 9.6). Between each step of the DAS-ELISA procedure, plates were washed 3 times with PBST buffer (137 mM NaCl, 8 mM Na_2_HPO_4_, 12H_2_O, 2,7 mM KCl, 1.5 mM KH_2_PO_4_, 0.05% (*v*/*v*) Tween 20, 2% (*w*/*v*)). Then, 100 µL of plant sap was added into coated wells and incubated overnight at 4 °C. Alkaline phosphatase conjugated-PAV52 antibody (100 µL) diluted (1/1000 (*v*/*v*)) in conjugated buffer (PBST buffer supplemented with 2% (*w*/*v*) polyvinylpyrrolidone 40T and 2% (*w*/*v*) ovalbumin) was deposited in wells and incubated for 3 h at 37 °C. Wells were filled with 100 µL of diethanolamine (1N, pH = 9.8) containing p-nitrophenylphosphate (1 mg/mL). After incubation at room temperature in the dark for 1 h, optical density at 405 nm (OD_405_) was recorded for each well using a spectrophotometer (Multiskan™ FC; Thermo Scientific™, Waltham, MA, USA). A positive detection of BYDV-PAV in a tested sample was considered when the OD_405_ value was more than twice the OD_405_ value obtained for healthy control samples, with a minimum value of OD_405_ = 0.1.

### 2.4. Statistical Analyses

To investigate aphid colonization and virus spread in the arena, several parameters linked to the infestation and infection of tillers were collected and analyzed with different statistical tests.

#### 2.4.1. Population Scale Analysis

The effect of the founder aphid *morph* and the clover *treatment* on different variables was analyzed at the population/arena scale (i.e., 1 data per arena). The variables were the distance founder tiller–deposit tiller, aphids per arena, aphids per founder tiller, the occurrences of aphids and virus per tiller, and virus occurrence per non-infested tiller and per infested tiller. Generalized Linear Mixed Effects Models (GLMM) were used with a *session* random effect on the intercept to account for potential heterogeneity in aphid population growth and virus transmission between experiments. The model included the fixed effect of the *morph* (2-level factor: wingless and alate morphs) in interaction with the fixed effect of the *treatment* (3-level factor: b, bc1 and bc2).

Variable tested ~ *morph* + *treatment* + *morph* × *treatment* + (1|*session*)

A gaussian link was used for distance founder tiller–deposit tiller after a square root transformation, and a binomial family link was used for occurrence variables and a negative binomial link for count variables to account for overdispersion. The significance of fixed factors and their interaction was determined by a likelihood ratio test (LRT).

#### 2.4.2. Tiller Scale Analysis

The effect of the clover *treatment* and the *distance* from the founder tiller on different variables was analyzed at the tiller scale (i.e., 1 data per tiller). The data were analyzed separately considering the two founder aphid morphs. The variables were aphids per tiller or per infested tiller, the occurrences of aphids and virus per tiller, and the occurrences of virus per infested and non-infested tiller. To analyze the spatial spreading pattern of variables around the founder tiller, the distance from the founder tiller (numerical) was used as an explanatory variable. GLMM with a binomial link were used for occurrence variables and GAMM (Generalized Additive Mixed Effects Models) with a negative binomial link for count data that displayed non-linear responses regarding to the distance variable. The model included the fixed effect of the *treatment* (3-level factor: b, bc1 and bc2) in interaction with the *distance* from the founder tiller (number of tillers from the founder tiller). The random effect of the *arena* nested in the *session* was used to account for non-independent observations due to experimental variations of aphid population growth and virus transmission.

Variable tested ~ *treatment* + *distance* + *treatment* × *distance* + (1|*session*/*arena*)

Statistical analyses were performed using R version 4.1.1 with the lme4 package for GLMM and gamm4 package for GAMM. The drop1 function was used to perform LRT.

## 3. Results

### 3.1. Morph and Virus Transmission

In the 65 arenas (distributed between the 3 treatments, Table 1), the founder nymphs became alate adult in 40 arenas (61.5%) and wingless adult in 25 arenas (38.5%). During the 13 days of the experiment, two generations of aphids were produced from the founder aphid. These generations were only composed of wingless individuals whatever the morph of the founder aphid. The tiller on which the founder aphid has established the first colony, i.e., the founder tiller, hosted 72.2 ± 3.7 aphids while other tillers hosted 5.4 ± 0.2 individuals. According to the viruliferous status of the founder aphid, plants (at least some of them including the founder tiller) of the arenas were expected to be infected by the BYDV-PAV4 isolate. However, analyses of the sanitary status of plants showed that infected plants were present in only 46 arenas (70.8%), suggesting that nymphs were not all viruliferous or didn’t have enough virus particles to reach the infection threshold. Thus, arenas without infected plants were removed from data set used to analyze viral spread.

For interactions between treatments (b, bc1 and bc2) and aphid morphs (wingless and alate), no significant statistical link was found, neither for the 65 arenas (*p* = 0.565) nor the 46 infected arenas (*p* = 0.351, Pearson’s Chi-squared test based on 10^6^ replicates). Thus, no confounding effects are expected between these factors in the following results.

### 3.2. Morph and Clover Effect at Population Scale

**Figure 2:**
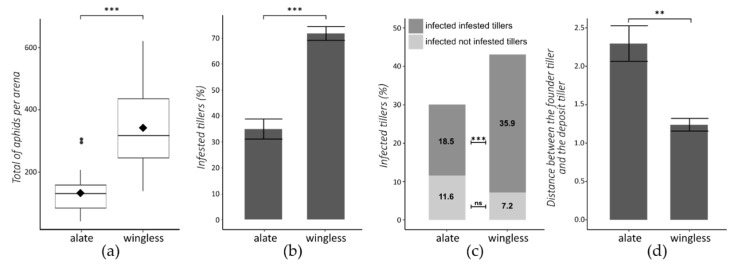
Aphid population and virus spread at the population scale, according the founder aphid morph. (**a**) Total number of aphids per arena (the black diamond represents the mean). (**b**) Percentage of tillers infested (Mean ± SEM); (**c**) percentage of tillers infected; (**d**) distance between the founder tiller and the deposit tiller. GLMM were used (see Section 2.4.1 for further details) **: *p* < 0.01, ***: *p* < 0.001, ns: not significant.

Analyses carried out at the population scale (1 data per arena) showed that clover has no significant effect on parameters linked to aphid colonization and virus spread (Table 2). However, there is a strong interaction between the morph and the treatment for all monitored parameters except for aphids per founder tiller and virus occurrence per non-infested tiller. For the wingless morph, the presence of clover in barley arenas tends to decrease size of aphid populations and rate of virus infections, whereas for the alate morph, these variables tend to increase (Table 2). For all parameters except virus occurrence per non-infested tillers, morph has a highly significant effect. Thus, for arenas started with a founder aphid with a wingless morph, the size of aphid populations is 2.6 times higher (Table 2 line c and Figure 2a), the number of aphids per founder tiller is 1.7 times higher (Table 2 line b, not illustrated), and the aphid occurrence per tiller is 2.0 times higher (Table 2 line d, Figure 2b) compared to arenas started with an alate founder morph.

To describe the spread of BYDV-PAV in the arena, the sanitary status of plants must be analyzed together with the presence/absence of aphids on each of the 36 plants of the arena. Infection rate in the whole arena (virus occurrence) and infection rate associated to infested plants (virus occurrence per infested tiller, i.e., hosting aphids) were significantly lower for arenas with an alate founder morph (Table 2 line e, Table 2 line g and Figure 2c). Virus occurrence per non-infested tillers is not significantly different between the two aphid morphs (Table 2 line f and Figure 2c). Concerning the distance between the founder tiller (tiller on which the founder aphid has established the first colony) and the deposit tiller (tiller on which the founder aphid was deposited at the beginning of the experiment, i.e., the tiller located at the row 3 and line 3), which can reflect the early mobility of the founder aphid before it settled on a tiller, the morph has a significant effect. For arenas started with an alate morph, this distance is twice the distance observed for arenas with a wingless founder morph (Table 2 line a and Figure 2d). Moreover, there is a slightly significant interaction between the morph and the treatment, probably explained by a distance founder tiller-deposit tiller halved for bc2 in case of alate founder morph (Table 2 line a).

### 3.3. Clover and Distance Effect at Tiller Scale

To complete the description of data associated to aphid density and virus occurrence in arenas, spatial parameters have been used to evaluate the impact of clover on the overall movement of the founder aphid and its progeny, and on the viral spread. Analyses were carried out at the tiller scale (36 data per arena).

**Table 3:** Aphid colonization and virus spread at the tiller scale.

		Variable		Fixed Factors		Wingless			Alate	
						*p*-Value	R^2^		*p*-Value	R^2^
	**Aphids**	occurrence per tiller		Distance		***p* < 0.001**	R^2^m: 0.07 R^2^c: 0.18		***p* < 0.001**	0.16
(a)				Treatment		**0.03**			0.33	
				D × T		0.96			***p* < 0.001**	
		per infested tiller		Distance		***p* < 0.001**	0.57		***p* < 0.001**	0.51
(b)				Treatment		**0.04**			0.15	
				D × T		0.21			0.26	
		per tiller		Distance		***p* < 0.001**	0.55		***p* < 0.001**	0.42
(c)				Treatment		**0.004**			0.42	
				D × T		0.10			**0.02**	
										
	**Virus**	occurrence per tiller		Distance		***p* < 0.001**	R^2^m: 0.03 R^2^c: 0.46		***p* < 0.001**	R^2^m: 0.06 R^2^c: 0.19
(d)				Treatment		0.81			0.79	
				D × T		0.57			0.10	
		occurrence per non-infested tiller		Distance		0.066	0.008		***p* < 0.001**	0.008
(e)				Treatment		0.57			0.61	
				D × T		0.81			0.90	
		occurrence per infested tiller		Distance		**0.004**	0.02		***p* < 0.001**	0.13
(f)				Treatment		0.76			0.76	
				D × T		0.40			0.07	

D: Distance to the founder tiller; T: treatment (b, bc1, bc2); D × T: interaction between distance and treatment. For each variable tested, GLMM (for occurrence variables) or GAMM (for count data that displayed non-linear responses regarding to the distance variable) were used including in the models the fixed effects of the treatment, the distance, the interaction between the two, and the arena nested in the session as a random effect (see Section 2.4.2 for further details). GLMM analysis: R^2^m: marginal R-squared value; R^2^c: conditional R-squared value. GAMM analysis: R^2^: adjusted R-squared value.

#### 3.3.1. Wingless Founder Morph

Concerning arenas started with a founder aphid with a wingless morph, the spatial distribution of aphids and of infected barley plants present a ‘one focus’ pattern, concentrated around the founder tiller. Indeed, the distance to the founder tiller has a high significant effect for all the studied parameters, except the virus occurrence per non-infested tiller (Table 3). With the distance to the founder tiller increased, the colonization of aphids (aphid occurrence per tiller, aphids per infested tiller, and aphids per tiller) and the spread of the virus (virus occurrence per tiller and virus occurrence per infested tiller) decreased.

**Figure 3:**
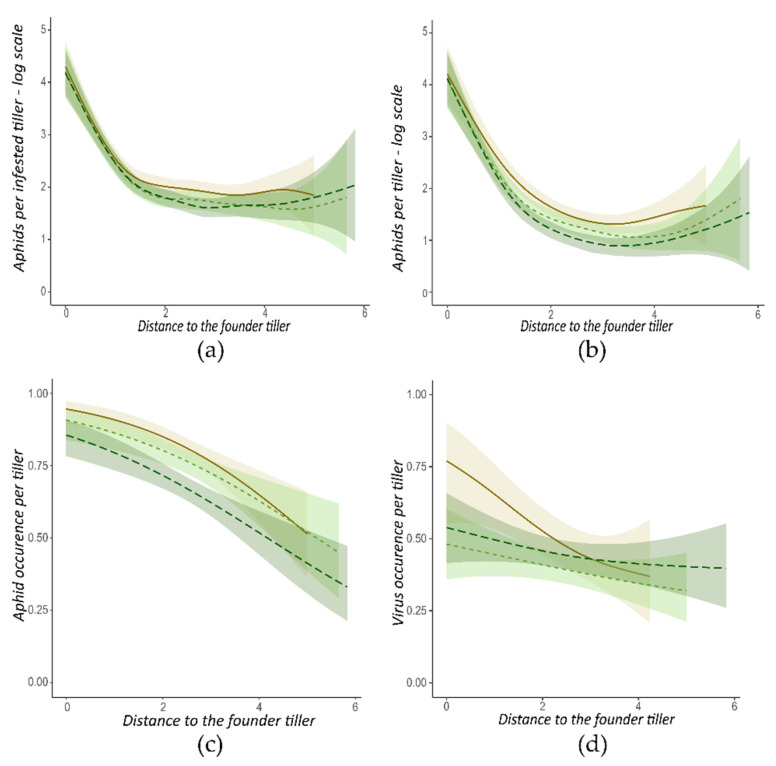
Aphid colonization and virus spread at the tiller scale, according to the distance from the founder tiller. Data from arenas started with a wingless founder morph are presented. (**a**) Log(Number+1) of aphids per infested tiller; (**b**) Log(Number+1) of aphids per tiller; (**c**) aphid occurrence per tiller; (**d**) virus occurrence per tiller. Each curve corresponds to one treatment: 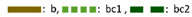. Areas are the 0.95 margin error for each curve.

The presence of clover in the arena reduces the size of aphid populations. Indeed, the treatment had a significant effect on aphids per infested tiller (Table 3 line b and Figure 3a), aphids per tiller (Table 3 line c and Figure 3b), and aphid occurrence per tiller (Table 3 line a and Figure 3c). When the clover density increases, the effect of clover is amplified for aphid occurrence per tiller (6% reduction for bc1 and 18% for bc2, compared to barley alone) and for aphids per tiller (21% reduction for bc1 and 27% for bc2). Concerning aphids per infested tiller, the effect of clover is similar for the two clovers densities (16% reduction for bc1 and 12% for bc2).

Although clover reduces the size of aphid populations in the arena, there is no effect of clover detected on the virus spread. Indeed, the treatment had no significant effect (*p*-values > 0.57) on virus-linked parameters (Table 3 line d, Table 3 line e, Table 3 line f, and Figure 3d).

Finally, the clover has no spatial effect on aphids and virus spread. It did not modify the ‘one focus’ pattern. Indeed, the interaction between distance and treatment was not significant for all the variables studied (Table 3).

#### 3.3.2. Alate Founder Morph

Concerning arenas started with a founder aphid with an alate morph, the distance to the founder tiller had a high significant effect for all monitored parameters. As the distance to the founder tiller increased, colonization of arena by aphids and the proportion of infected plants decreased (Table 3). In contrast with observations made for arenas associated to wingless founder morph, the presence of clover has no effect on aphid population size and on the spread of virus in arenas started with an alate founder morph (Table 3).

**Figure 4:**
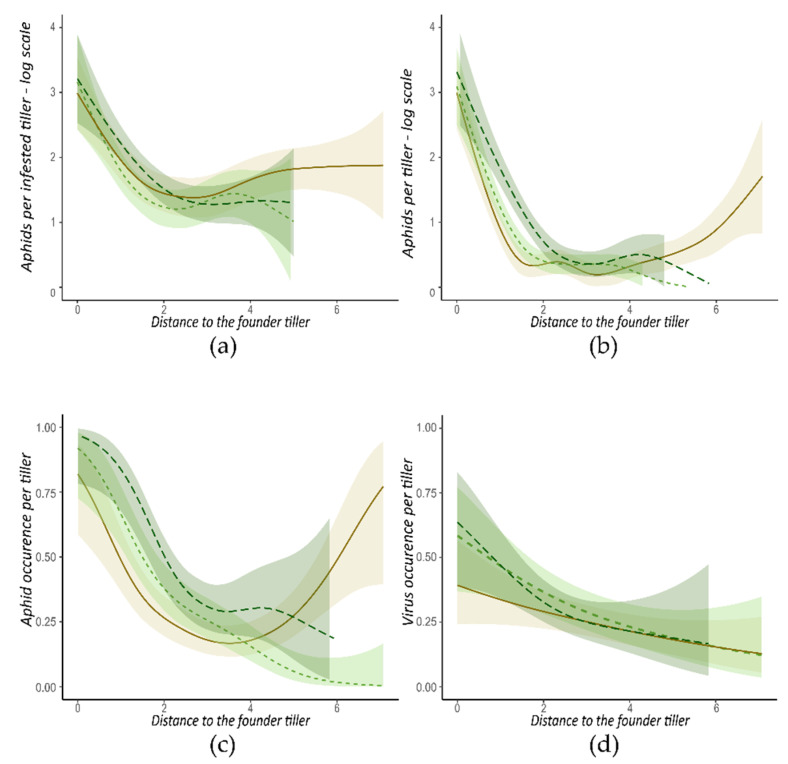
Aphid colonization and virus spread at the tiller scale, according to the distance from the founder tiller. Data from arenas started with an alate founder morph are presented. (**a**) Log(Number+1) of aphids per infested tiller; (**b**) Log(Number+1) of aphids per tiller; (**c**) aphid occurrence per tiller; (**d**) virus occurrence per tiller. Each curve corresponds to one treatment: 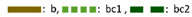. Areas are the 0.95 margin error for each curve.

The clover has a negative effect on the spatial distribution of aphids. Indeed, the interaction between the distance and the treatment had a significant effect on aphids per tiller (Table 3 line c and Figure 4b) and on aphid occurrence per tiller (Table 3 line a and Figure 4c). According to the ‘one focus’ shape described above, for arenas bc1 and bc2, aphid occurrence and aphids per tiller decreases when distance to the founder tiller increases, until these parameters are almost null for the most distant tillers (Figure 4b,c). However, for b arenas, these two parameters decrease up to a distance of 4 tillers away from the founder tiller and then increase until it reaches an aphid occurrence of 0.8 and 3.7 aphids per tiller for the most distant tillers (Figure 4b,c). This horseshoe-shaped curve suggested the development of two distant aphid colonies, i.e., a ‘two foci’ distribution, probably resulting from a “long distance” movement of the alate founder morph in the arena at an early step of the experiment.

For virus-linked parameters, the interaction between distance and treatment was not significant (Table 3 line d, Table 3 line e, and Table 3 line f). The clover, therefore, did not modify the shape of virus spread pattern, but a tendency for higher proportion of infected plants close to the founder tiller in the presence of clover is observed (Figure 4d).

## 4. Discussion

The introduction and spread of insect-borne viral disease in a field is mainly based on biological parameters of virus–plant interactions. However, the vector, through its interactions with the host plant and with the virus, plays also a major role in this process. Indeed, it will determine both efficiency and dynamics of plant-to-plant virus transmission. In the absence of a chemical treatment directly against viruses and in an agronomic context that lacks genetic solutions with resistant crop varieties to efficiently control the disease in fields, chemical solutions targeting vectors of plant viruses have often been preferred. In order to develop agroecological methods to control viral diseases, the impact of a non-host plant (i.e., clover plants) on the biological and spatial parameters of the epidemiology of barley yellow dwarf disease was, therefore, tested.

Our experiment with barley plantlets and conducted with or without the presence of clover did not reveal an overall general benefit of clover on the dynamics of aphid populations nor on the disease spread. However, the analysis, by separating the two founder morphs, shows differentiated effects of the non-host clover on parameters linked to the epidemiology of the aphid-transmitted yellow dwarf disease.

The initial step of field colonization by aphids is the introduction of alate individuals from wild or cultivated reservoirs located outside the field [29,30]. When these individuals reach a field grown with their host plant, alate aphid land, probe, and fly between several plants before settling down in a more sedentary manner [31]. We observed, through our measurements in the arenas with barley alone, distribution patterns that could well be explained by these “trivial flights”. Indeed, several founder sites of aphids were observed within the arenas initiated with an alate founder morph. This initial mobility of founder alate individuals is altered in our experimental set-up by the presence of clover between barley plants, which limits the number of aphid founder sites to one. Another parameter shows that alate mobility is altered in the presence of clover in our set-up. Indeed, the distance founder tiller-deposit tiller is reduced by 2.5 for bc2 arenas compared to barley alone. The first day of the experiment, the clover was at the cotyledon stage, and it developed to reach the 1-leaf stage at the time the founder aphid reached the adult stage (not illustrated). Thus, when the alate individuals start plant-to-plant movements in the arena, the clover is in average 5 cm high. It is surprising that the spatial dynamics of the aphids can be altered by the presence of these small non-host plants, since alate can fly without being disturbed by the physical barrier that the clover might represent at this step of the experiment. It cannot be ruled out that, under our experimental conditions, the movements of the alate aphids from multiple founder sites occurs through ground movements by walking on the soil form plant-to-plant. However, the clover plantlets would also participate to the modification of the environment at olfactory and/or visual levels, disturbing spatial movement of alate founder *R. padi.* Thus, based on data collected from arena design, the process of introduction of alate aphids in barley fields, which is one of the most important steps for the incidence of YDD at field scale (i.e., intensity of primary inoculations), could be spatially disturbed by the presence of a non-host plant such as clover, reducing the number of founder sites per migrant alate aphid.

From these founder sites initiated by alate individuals in the field, several generations of wingless aphids will develop by parthenogenesis, increasing the size of the founder site by colonizing neighboring plants [32]. Population density is known to be an important parameter in the colonization of space by aphids. The presence of clover in our work showed a significant decrease in several descriptive parameters of aphid populations generated from a wingless founder morph. Indeed, the number of aphids per infested tiller and the percentage of plants infested were reduced in the presence of clover. This result is consistent with other studies testing the effect of plant diversification on aphid populations [33]. The non-host plant can disturb aphid behavior by different mechanisms. In the presence of clover, aphids may spend more time on the non-host plant at the cost of the host plant, leading to a decrease of the total size of the produced population. When the founder aphid has an alate morph, we do not observe a reduction of the following aphid populations in the presence of clover. However, it is important to note that the total number of aphids produced in an arena by alate founder morph was 2.6 times lower than the population produced by wingless founder morph. It has been reported that the two aphid morphs present different life history traits, especially a longer nymphal development and a lower offspring production for alate aphids [34]. Khan and Port [35] reported a fecundity of alate *R. padi* reduced by half compared to wingless adult. The effect of clover on *R. padi* abundance may be visible only with high-density populations, as shown in a previous study on genetic plant diversification [36], which was not the case under our experimental conditions with alate aphids. Then, the spatial effect of the clover observed in arenas initiated by an alate founder morph was not observed in the case of the wingless morph. Since in both morphs the following generations in arenas are wingless aphids, the spatial effect of the clover acts only on the founder individual. This absence of spatial effect of clover can be explained by the low mobility of the wingless adult, supported by the distance founder tiller-deposit tiller measurement, which is on average only 1 tiller. Thus, the observations made in this study show that the dynamics of extension of the initial infestation sites, ensured by the migration from plant-to-plant of wingless individuals, is partly lowered by the presence of clover.

Despite the effect of clover on the abundance of aphids (founder wingless morph) or on the distribution of aphids (founder alate morph), we did not detect an effect of the clover on the BYDV incidence. Vector abundance is obviously an important parameter in the epidemiology of viral disease. However, as described in numerous studies, vector behavior (i.e., feeding behavior or dispersal behavior) should be considered as one of the key parameters for virus spread [37,38]. The efficiency of plant-to-plant virus transfer depends on both the efficiency of acquisition of viral particles from an infected plant and the inoculation of virus to a healthy host. These two steps rely on characteristics of feeding behavior of the insect vector. This feeding behavior could be modified in an intercropping system compared to a monoculture one. For example, it has been shown that leafhoppers, which are plant sucking insects, spent more time on their host plant in a genetically diversified crop system, leading to an increase in the spiroplasma transmission rate [39]. Thus, further studies are needed to understand whether the non-homogeneous environment constituted by the association of barley and clover may modify *R. padi* feeding behavior. Concerning the dispersal behavior of insects in a diversified habitat, the non-host plant could increase insect mobility, increasing the number of plants visited and infected. In our experiment, the virus occurrence per non-infested tiller allowed us to test this mobility at the population scale. This parameter remains unchanged in the presence of clover, whatever the morph of the founder aphid.

In a plant-insect-virus pathosystem, the virus can also influence the insect behavior. Concerning *R. padi* and BYDV, several studies showed that virus-free *R. padi* were more attracted by infected plants [40,41,42], particularly by the VOCs emitted by these plants [43], while viruliferous *R. padi* preferred healthy hosts [41,42]. This is an example of the “vector manipulation hypothesis”, commonly found for insect-borne viruses [44]. This vector manipulation can explain why *R. padi* abundance is decreased in our experiments in the presence of clover, but not the BYDV incidence. Finally, it cannot be excluded that clover has an impact on BYDV but that the effect was not observed under our experimental design.

The arena-based experiment carried out using barley/clover associations gives indications regarding the presence of clover and the ability of *R. padi* to colonize cultivated barley fields. Growing these two plant species together would reduce (i) the number of founder sites initiated by alates for the development of offspring, (ii) the number of infested barley plants by wingless aphids, and (iii) the size of aphid colonies. Thus, the presence of clover plants in barley growing areas would participate to lower the risks associated to the presence of BYDV vectors through a bottom-up regulation of aphid populations within the field. To better characterize if and how barley/clover intercropping would reduce BYDV prevalence, further laboratory experiments and field trials are needed.

## Figures and Tables

**Table 1:** Wingless and alate founder morph in arenas.

	Founder Aphid Morph *	
Treatment	Wingless	Alate
b	13 (6)	10 (7)
bc1	12 (11)	9 (5)
bc2	15 (12)	6 (5)

* Total number of arenas (Number of arenas containing infected plants).

**Table 2:** Aphid population and virus spread at the population scale.

		Variable		Wingless					Alate					*p*-Value				R^2^	
				All Arenas	b	bc1	bc2		All Arenas	b	bc1	bc2		M	T	M × T		R^2^m	R^2^c
(a)		Distance founder tiller–deposit tiller		1.2 ± 0.1	1.1 ± 0.1	1.2 ± 0.1	1.4 ± 0.1		2.3 ± 0.2	2.8 ± 0.3	2.5 ± 0.4	1.1 ± 0.4		**0.001**	0.11	***p* < 0.001**		0.19	0.19
(b)	**Aphids**	per founder tiller		85.4 ± 3.9	93.4 ± 6.2	79.7 ± 8.3	83.0 ± 6.0		51.1 ± 4.9	42.7 ± 8.0	61.4 ± 8.8	49.5 ± 6.8		***p* < 0.001**	0.92	0.22		0.29	0.29
(c)		per arena		342.2 ± 21.6	412.9 ± 39.1	316.3 ± 32.2	301.7 ± 34.6		132.7 ± 13.5	112.4 ± 18.4	137.9 ± 23.4	158.7 ± 32.4		***p* < 0.001**	0.44	**0.009**		0.53	0.63
(d)		occurrence per tiller (%)		72.4 ± 2.7	80.5 ± 3.0	70.8 ± 5.7	66.7 ± 4.3		35.2 ± 3.9	28.6 ± 4.3	36.1 ± 8.7	44.9 ± 6.3		***p* < 0.001**	0.41	***p* < 0.001**		0.66	0.85
																			
(e)	**Virus**	occurrence per tiller (%)		43.1 ± 5.8	50.5 ± 13.2	37.1 ± 7.7	44.9 ± 10.5		29.9 ± 3.9	24.6 ± 5.7	36.1 ± 7.5	31.1 ± 8.2		***p* < 0.001**	0.57	**0.01**		0.38	0.62
(f)		occurrence per non-infested tillers (%)		7.2 ± 1.5	7.9 ± 2.8	7.3 ± 2.5	6.7 ± 2.5		11.6 ± 1.3	11.1 ± 2.5	13.9 ± 9.9	9.4 ± 3.2		0.11	0.39	0.78		0.06	0.33
(g)		occurrence per infested tillers (%)		35.9 ± 4.9	42.6 ± 11.6	29.8 ± 6.1	38.2 ± 8.9		18.5 ± 3.3	13.5 ± 3.2	22.2 ± 6.9	21.7 ± 7.8		***p* < 0.001**	0.28	***p* < 0.001**		0.56	0.66

b: barley; bc1: barley-clover density 1; bc2: barley-clover density 2; M: morph of the founder aphid (wingless, alate); T: treatment (b, bc1, bc2); M × T: interaction between morph and treatment. Mean ± SEM of all arenas combined according to the morph of the founder aphid, mean ± SEM by treatment. For each variable tested, GLMM were used, including in the models the fixed effects of the morph, the treatment, the interaction between the two, and the session as a random effect (see Section 2.4.1 for further details). Results of the statistical test are shown. R^2^m: marginal R^2^ value considering only the variance explained by the fixed effects; R^2^c: conditional R^2^ value considering the variance explained by both fixed and random effects.

## Data Availability

The data presented in this study are available on request from the corresponding authors.
